# The impact of gender, puberty, and pregnancy in patients with POLG disease

**DOI:** 10.1002/acn3.51199

**Published:** 2020-09-18

**Authors:** Omar Hikmat, Karin Naess, Martin Engvall, Claus Klingenberg, Magnhild Rasmussen, Chantal M. E. Tallaksen, Christian Samsonsen, Eylert Brodtkorb, Elsebet Ostergaard, Rene de Coo, Leticia Pias‐Peleteiro, Pirjo Isohanni, Johanna Uusimaa, Niklas Darin, Shamima Rahman, Laurence A. Bindoff

**Affiliations:** ^1^ Department of Paediatrics and Adolescent Medicine Haukeland University Hospital Bergen 5021 Norway; ^2^ Department of Clinical Medicine (K1) University of Bergen Bergen Norway; ^3^ Centre for Inherited Metabolic Diseases Karolinska University Hospital Stockholm Sweden; ^4^ Department of Medical Biochemistry and Biophysics Karolinska Institute Stockholm Sweden; ^5^ Department of Molecular Medicine and Surgery Karolinska Institute Stockholm Sweden; ^6^ Department of Paediatric and Adolescent Medicine University Hospital of North Norway Tromso Norway; ^7^ Paediatric Research Group Department of Clinical Medicine UiT‐ The Arctic University of Norway Tromso Norway; ^8^ Women and Children's Division Department of Clinical Neurosciences for Children Oslo University Hospital Oslo Norway; ^9^ Unit for Congenital and Hereditary Neuromuscular Disorders Department of Neurology Oslo University Hospital Oslo Norway; ^10^ Department of Neurology Oslo University Hospital Oslo Norway; ^11^ Institute of Clinical Medicine Faculty of Medicine University of Oslo Oslo Norway; ^12^ Department of Neuroscience Norwegian University of Science and Technology Trondheim Norway; ^13^ Department of Neurology and Clinical Neurophysiology St. Olav's University Hospital Trondheim Norway; ^14^ Department of Clinical Genetics Copenhagen University Hospital Rigshospitalet Copenhagen Denmark; ^15^ Department of Neurology Medical Spectrum Twente Enschede The Netherlands; ^16^ Department of Genetics and Cell Biology University of Maastricht Maastricht The Netherlands; ^17^ Department of Neurology Sant Joan de Déu Children´s Hospital Barcelona Spain; ^18^ Department of Pediatric Neurology Children's Hospital Helsinki University Hospital Helsinki Finland; ^19^ Stem Cells and Metabolism Research Program Faculty of Medicine University of Helsinki Helsinki Finland; ^20^ PEDEGO Research Unit University of Oulu Oulu Finland; ^21^ Department of Pediatric Neurology Clinic for Children and Adolescents Medical Research Center Oulu University Hospital Oulu Finland; ^22^ Department of Pediatrics The Queen Silvia Children's Hospital University of Gothenburg Gothenburg Sweden; ^23^ Mitochondrial Research Group UCL Great Ormond Street Institute of Child Health London United Kingdom; ^24^ Metabolic Unit Great Ormond Street Hospital for Children NHS Foundation Trust London United Kingdom; ^25^ Department of Neurology Haukeland University Hospital Bergen 5021 Norway

## Abstract

**Objective:**

To study the impact of gender, puberty, and pregnancy on the expression of POLG disease, one of the most common mitochondrial diseases known.

**Methods:**

Clinical, laboratory, and genetic data were collected retrospectively from 155 patients with genetically confirmed POLG disease recruited from seven European countries. We used the available data to study the impact of gender, puberty, and pregnancy on disease onset and deterioration.

**Results:**

We found that disease onset early in life was common in both sexes but there was also a second peak in females around the time of puberty. Further, pregnancy had a negative impact with 10 of 14 women (71%) experiencing disease onset or deterioration during pregnancy.

**Interpretation:**

Gender clearly influences the expression of POLG disease. While onset very early in life was common in both males and females, puberty in females appeared associated both with disease onset and increased disease activity. Further, both disease onset and deterioration, including seizure aggravation and status epilepticus, appeared to be associated with pregnancy. Thus, whereas disease activity appears maximal early in life with no subsequent peaks in males, both menarche and pregnancy appear associated with disease onset or worsening in females. This suggests that hormonal changes may be a modulating factor.

## Introduction

Mitochondria are essential organelles found in almost all human cells. The most fundamental function of mitochondria is energy production through the process of oxidative phosphorylation (OXPHOS).^1^ The respiratory chain conserves the energy released through intermediary metabolism and the ATP synthase then uses it to generate adenosine triphosphate (ATP). Thirteen of the protein subunits of the OXPHOS system are encoded by mitochondrion’s own DNA (mtDNA), while all the remaining subunits are encoded by the nuclear DNA (nDNA).^2^ Mitochondrial dysfunction can present at any age with heterogeneous clinical manifestations and multi‐organ involvement. Nevertheless, mitochondrial disease appears predominantly to affect tissues with high energy requirement, such as the brain, liver, and heart.^3^, ^4^

Polymerase gamma is the enzyme responsible for replicating and repairing mtDNA. Variants in *POLG*, the nuclear gene encoding the catalytic subunit of this enzyme, are among the most frequent causes of mitochondrial disease^5^, ^6^ and cause a variety of clinical phenotypes ranging from infantile refractory epilepsy and liver failure to juvenile and adult‐onset epilepsy, myopathy and ataxia, and to late‐onset myopathies with progressive external ophthalmoplegia.^7^, ^8^, ^9^, ^10^ Epilepsy has a major impact on morbidity and mortality. Data from a large cohort of individuals with POLG disease, which was recently published, showed that epilepsy is significantly associated with poor outcome.^11^

Mitochondrial diseases have a minimum prevalence of ca. 1:5000.^12^ Despite the fact that they are considered the most common form of inborn error of metabolism, information on the impact of gender on these diseases remains limited and stems mainly from case reports and small case‐series of pregnant women with disorders, such as mitochondrial encephalopathy, lactic acidosis, and stroke‐like episodes (MELAS).^13^, ^14^, ^15^, ^16^, ^17^, ^18^ Since mutations in *POLG* are one of the most common causes of mitochondrial disease, we wanted to study the impact of gender, puberty, and pregnancy on POLG disease with the aim of providing insights to guide physicians responsible for their management.

## Patients and Methods

### Study design and population

We conducted a multicenter retrospective study of 155 patients with confirmed pathogenic *POLG* variants from 12 centers in seven European countries: Norway, Finland, Sweden, Denmark, The Netherlands, Spain, and the United Kingdom. Detailed demographic, clinical, laboratory, neuroimaging, and genetic data were obtained. Age of disease onset was defined as age in months when the patient first required medical evaluation. The age of onset of each individual symptom was also identified. End of follow‐up was defined as the patient’s last visit to the hospital or death. Secondary amenorrhea was defined as cessation of menstruation for six consecutive months in a woman who has previously had regular menstruation. A detailed description of the clinical, laboratory, and neuroimaging data for the whole cohort has been published.^11^ The mean age of thelarche and menarche in the population of each country participating in the study^19^, ^20^, ^21^, ^22^, ^23^, ^24^, ^25^, ^26^, ^27^ is provided in File S1.

### Data analysis

In order to study the effect of gender, individuals were stratified into two groups according to their gender and further grouped into those with disease onset before and after onset of puberty (12 years). To study the effect of pregnancy, females of childbearing age (18–50 years) were identified and their data were analyzed separately.

### Statistical analysis

Statistical analysis was performed using SPSS (Statistical Package of Social Sciences), Version 23.0. A two‐sided *P* value less than 0.05 was considered to be statistically significant. For survival analysis, the end‐point was time to death, which was defined as the time in months from the date of disease onset to the date of death. Univariate survival analysis was performed using log‐rank test (Kaplan–Meier) to compare differences in survival time between the subgroups defined above.

### Ethical statement

The ethical approval for the study was obtained from the Regional Committee for Medical and Health Research Ethics, Western Norway (REK 2014/1783‐4). Each participating country had obtained approval by the local ethical committee. The study was registered as an audit at Great Ormond Street Hospital, London, UK (Registration Number 1675).

## Results

### Demography

In the whole study cohort of 155 individuals, age of disease onset extended from birth to 71 years with median age of onset 10 years (interquartile range: 19 years). Fifty‐four percent of individuals (*n* = 83) had disease onset before the age of 12 years and 46% (*n* = 72) after this age. Fifty‐one percent (*n* = 79) were females and 49% (*n* = 76) were males.

### Major clinical features and gender differences

**Figure 1:**
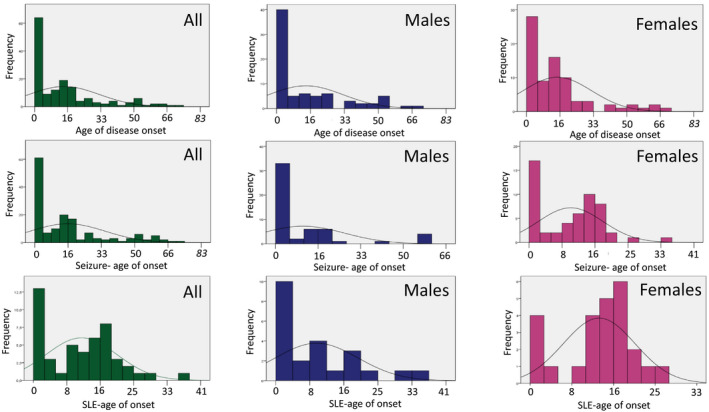
Age of disease onset, onset of seizure, and onset of stroke‐like episodes (SLE) in years for the whole study cohort of individuals with POLG disease and also according to the gender. The figure shows clearly that the age of disease onset, seizure and stroke‐like episode (SLE) onset in females had a bimodal distribution with the first peak in childhood and the second peak around puberty, while males were presented earlier, and the age of disease onset, seizures and SLE onset had a unimodal distribution with peak of onset during the childhood.

Median age of disease onset for males was 5 years, while for females it was 13 years. Further, the percentage of males and females presenting prior to or after the age of 12 years differed; before age 12, we found 55% males (*n* = 46) and 45% females (*n* = 37) while after 12 years, it was 42% (*n* = 30) and 58% (*n* = 42). When we analyzed age of onset for the whole cohort, we found a bimodal distribution with the first peak early in life (before the age of 4 years) and the second around 12–16 years. This bimodal pattern was, however, gender dependent; it was apparent only in females and was not seen when we analyzed data only from males (Fig. 1).

Seizures occurred in 69% (*n* = 107), and acute episodes of worsening (previously called stroke‐like episodes (SLE)) in 37% (*n* = 52) of individuals. Once again there was a trend toward a gender difference; a bimodal distribution with one peak in early life and the second around puberty was seen in females but not males for both seizures and SLE (Fig. 1). Further, regardless of the age of disease onset, status epilepticus was reported in 56% (*n* = 44/79) of females and 44% (*n* = 35/79) of males.

**Figure 2:**
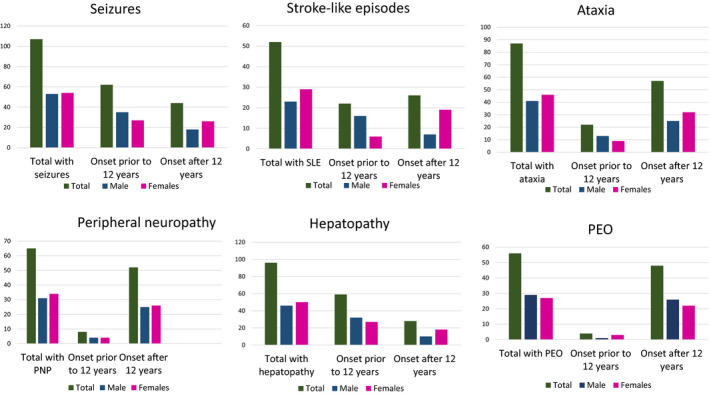
Major clinical features of POLG patients stratified according to gender and puberty onset. SLE, stroke‐like episodes, PEO, progressive external ophthalmoplegia, PNP, peripheral neuropathy.

When we looked at other features of POLG disease, such as ataxia and liver dysfunction, these manifested more often in males prior to the age of 12 years than females under 12, while in those with disease onset after the age of 12 (Fig. 2), it was the reverse. Features such as peripheral neuropathy, progressive external ophthalmoplegia, and ptosis did not show any clear gender differences. A detailed description of the frequency of each individual symptom before and after the onset of puberty is provided in Figure 2 and in File S2.

Secondary amenorrhea was reported in three patients (one homozygous for p.Trp748Ser, one compound heterozygous for p.Trp748Ser/p.Gln497His, and one heterozygous for the dominant p.Tyr955Cys).

### Impact of pregnancy

Our cohort included 14 women between 18 and 50 years whom we considered potentially fertile and of childbearing age. Ten of these became pregnant and in all, pregnancy had a clear negative impact: five patients presented with the first signs of their disease in pregnancy and five with a clinical deterioration. The most critical period for these women appeared to be the second trimester of pregnancy (Table 1). Pregnancy was associated with status epilepticus in six individuals and in three there was evidence of new‐onset liver dysfunction (study cases 6, 7, and 9).

**Table 1:** Summary of pregnancy data on POLG patients included in the current study.

Patient no.	Variants	Age at disease onset	Age at pregnancy	Parity^1^	Impact of pregnancy	Gestational age at disease debut or exacerbation in weeks	Symptoms at disease debut/exacerbation	Survival status	Age at death
1	p.Ala467Thr/p.Ala467Thr	18	18	G1P1	Debut	18	Seizure	Deceased	54
2	p.Ala467Thr/p.Ala467Thr	23	23	G1P1	Debut	20	Seizure	Alive	
3	p.Trp748Ser + p.Glu1143Gly^2^/p.Trp748Ser + p.Glu1143Gly^2^	19	33	G1P1	Exacerbation	19	Seizure/SE^3^	Alive	
4	p.Trp748Ser/p.Trp748Ser	15	20	G1P1	Exacerbation	17	Ataxia	Alive	
5	p.Gly268Ala/p.Gly268Ala	39	39	G1P1	Debut	16	Ataxia	Deceased	45
6	p.Trp748Ser/p.Trp748Ser	18	24	G3P2	Exacerbation	18	Seizure/SE^3^	Deceased	44
7	p.Ala467Thr/p.Ala467Thr	20	20	G1P1	Debut	19	Seizure/SE^3^	Deceased	39
8	p.Ala467Thr/p.Trp738Ser	14	23	G1P1	Exacerbation	32	Seizure/SE^3^	Deceased	24
9	p.Ala467Thr/p.Trp738Ser	27	27	G1P1	Debut	17	Ataxia	Alive	
10	p.Trp748Ser/p.Trp748Ser	18	18	G1P1	Debut	16	Seizure/SE^3^	Deceased	21

1. G: number of pregnancies, P: number of deliveries after 24 weeks. 2. Polymorphism. 3. SE: status epilepticus.

All women who experienced pregnancy‐associated disease onset/worsening were homozygous or compound heterozygous for the common founder variants p.Ala467Thr and p.Trp748Ser apart from one who was homozygous for the p.Gly268Ala variant (Table 1).

### Survival and gender differences

There was no significant difference in the overall survival between males and females. The analysis also showed that the onset of puberty did not have an impact on survival, regardless of gender. Interestingly, however, time to death was clearly shorter in females (median = 77 months) as compared to males (median = 108 months) regardless of the age of disease onset.

## Discussion

Our study shows that there are differences in the timing and in the manner that POLG disease manifests between males and females. Timing of disease onset showed a bimodal distribution in females with the first peak in childhood and the second around the time of puberty. Indeed, approximately half of the females in our cohort remained asymptomatic until they reached puberty. In addition, our study shows that pregnancy is a potential risk for women with POLG disease. Two thirds of women of childbearing age in our cohort either had disease onset or experienced disease deterioration during pregnancy. In contrast, the peak age of onset in males was unimodal and early in life.

The presenting feature of the disease also showed gender differences. Seizures, SLE, hepatopathy, and ataxia are major features of POLG disease and their presence reflects severity of the disease. In our cohort, seizures and SLE were more common in males prior to the onset of puberty, while in females, they appeared more common after the onset of puberty, particularly SLE. Hepatopathy and ataxia showed similar age‐related trends, albeit less dramatic. In contrast, the frequency of peripheral neuropathy and PEO showed similar gender frequencies before and after the age of puberty (Fig. 2, File S2).

Almost all the women who experienced pregnancy‐associated disease onset/worsening were homozygous or compound heterozygous for the common founder variants p.Ala467Thr and p.Trp748Ser; this may reflect ascertainment bias as all of these individuals were recruited from Norway where those variants are common^10^. Exacerbation of seizures was the major and predominant complication during pregnancy and was associated with status epilepticus, prolonged hospitalization, and the use of multiple antiepileptic drugs. Further, two of the individuals had disease onset manifesting with ataxia and one had a clear worsening of ataxia during pregnancy (Table 1).

The finding of secondary amenorrhea in three women in our cohort confirms previous reports that documented a link between pathogenic *POLG* variants and premature ovarian failure.^28^, ^29^

Changes in estrogen and progesterone levels clearly provide a link between menarche and pregnancy. Experimental animal models show that both hormones can alter neuronal excitability.^30^, ^31^ Estrogen increases the excitatory neurotransmitters and may alter the structure of the synaptic area by increasing the number of dendritic spines leading to increased cell‐to‐cell contact. Estrogen may also contribute to increased brain‐derived neurotrophic factor, which in turn may lead to excess glutamate release and decreased seizure threshold. Estrogen is therefore considered to be pro‐convulsant.^31^, ^32^ Progesterone appears to have a seizure inhibitory effect. Metabolites, such as allopregnanolone, a GABAA receptor‐modulating neurosteroid, appear to regulate hippocampal neuronal excitability by exerting a positive allosteric effect on GABAA receptors.^33^, ^34^ Studies have, moreover, demonstrated clustering of seizures in association with cyclic ovarian hormonal changes also in humans.^32^, ^35^ The estrogen peak occurring with puberty and the menstrual cycle may also explain the timing of disease onset or deterioration in females with POLG disease.

Pregnancy is a high‐demand metabolic state that requires optimal energy production to maintain normal physiological adaptation and fetal growth. Increased levels of serum lactate during pregnancy, even in healthy women,^36^ suggest, therefore, that pregnancy is associated with metabolic adaptions and potentially a degree of mitochondrial stress. It is possible to hypothesize that the increased energy demands affect all tissues, including neurons, and since these are already metabolically compromised, seizure threshold may be reduced. Our study showed that pregnancy is indeed a high‐risk period for women with POLG disease, particularly the second trimester.

Another potential risk factor is folate deficiency. This can result in mitochondrial dysfunction by reducing the expression of genes encoding essential proteins of the electron transport chain such as cytochrome c oxidase.^37^, ^38^, ^39^, ^40^, ^41^ The 10 pregnant women included in our study (Table 1) were Norwegian and would most probably have followed Norwegian pregnancy guidelines including the recommended daily supplement of 400‐microgram folate. This is, however, only in the first trimester (www.helsenorge.no). Thus, any potential protective effect given by folate supplementation in the first trimester may be lost following discontinuation and could contribute to the disease onset in the second trimester.

Given the results of our study, it is pertinent to ask whether women with POLG disease, especially those reaching childbearing age, should be counseled on the potential risk of disease deterioration during pregnancy. Clearly, proper planning and discussion of issues, such as sexual health, contraception, and pregnancy, are important. Nevertheless, our data suggest that there is a definite risk associated with pregnancy and that this is the case both in those with previously recognized disease and in those who first manifest their disease during pregnancy. Given this, we feel that women with POLG disease should be informed of the risks involved and, should they wish to proceed, we would suggest that they be managed both prior to and during pregnancy by a multispeciality team including obstetricians and neurologists/epileptologists.

Identifying those at greatest risk and, thus, those requiring maximal follow‐up is challenging. Individuals with POLG‐related epilepsy show EEG changes particularly involving the occipital regions, and these are present before the onset of clinically apparent seizures.^42^, ^43^, ^44^ While this is not a particularly robust biomarker, it may be useful in some patients. In the light of the second trimester worsening, the use of folic acid supplementation beyond the first trimester may also be a relevant option. Further studies will be needed to define the risk profile and show how best to manage fertile women with POLG disease; however, our data provide a clear indication that pregnancy and menarche are times of increased risk for females with *POLG* mutations.

## Conflict of Interest

The authors declare no financial or other conflict of interest.

## Authors Contributions

O.H and L.B designed the study, were responsible for data collection, analyzed the data and drafted the initial manuscript, and approved the final manuscript as submitted. K.N, M. E., C. K., M.R., C.ME. T., C.S, E.B., T.F., E.O., R. d.C, L.P., P.I., J.U., N.D., and S.R. were responsible for data acquisition and analysis, revising the manuscript critically, and approving the final manuscript as submitted. All authors are responsible for accuracy and integrity of the work.

## Supporting information


**File S1.** Timing of thelarche and menarche in the population of each participating country in the study.

**File S2.** Major clinical features stratified according to the gender and puberty onset.

## References

[1] Smeitink J , van den Heuvel L , DiMauro S . The genetics and pathology of oxidative phosphorylation. Nat Rev Genet 2001;2:342–352. 11331900 10.1038/35072063

[2] Spinazzola A , Zeviani M . Disorders from perturbations of nuclear‐mitochondrial intergenomic cross‐talk. J Intern Med 2009;265:174–192. 19192035 10.1111/j.1365-2796.2008.02059.x

[3] Schapira AH . Mitochondrial diseases. Lancet 2012;379:1825–1834. 22482939 10.1016/S0140-6736(11)61305-6

[4] Rahman J , Rahman S . Mitochondrial medicine in the omics era. Lancet (London, England) 2018;391:2560–2574. 10.1016/S0140-6736(18)30727-X 29903433

[5] Longley MJ , Graziewicz MA , Bienstock RJ , Copeland WC . Consequences of mutations in human DNA polymerase gamma. Gene 2005;18:125–131. 10.1016/j.gene.2005.03.029 15913923

[6] Rahman S , Copeland WC . POLG‐related disorders and their neurological manifestations. Nat Rev Neurol 2019;15:40–52. 30451971 10.1038/s41582-018-0101-0 PMC8796686

[7] Hudson G , Chinnery PF . Mitochondrial DNA polymerase‐gamma and human disease. Hum Mol Genet 2006;15:R244–R252. 16987890 10.1093/hmg/ddl233

[8] Saneto RP , Naviaux RK . Polymerase gamma disease through the ages. Dev Disabil Res Rev 2010;16:163–174. 20818731 10.1002/ddrr.105

[9] Hikmat O , Tzoulis C , Chong WK , et al. The clinical spectrum and natural history of early‐onset diseases due to DNA polymerase gamma mutations. Genet Med 2017;19:1217–1225. 28471437 10.1038/gim.2017.35

[10] Tzoulis C , Engelsen BA , Telstad W , et al. The spectrum of clinical disease caused by the A467T and W748S POLG mutations: a study of 26 cases. Brain 2006;129(Pt 7):1685–1692. 16638794 10.1093/brain/awl097

[11] Hikmat O , Naess K , Engvall M , et al. Simplifying the clinical classification of polymerase gamma (POLG) disease based on age of onset; studies using a cohort of 155 cases. J Inherit Metab Dis 2020;43:726–736. 32391929 10.1002/jimd.12211

[12] Skladal D , Halliday J , Thorburn DR . Minimum birth prevalence of mitochondrial respiratory chain disorders in children. Brain 2003;126(Pt 8):1905–1912. 12805096 10.1093/brain/awg170

[13] Wilcox G . Impact of pregnancy on inborn errors of metabolism. Rev Endocr Metab Disord 2018;19:13–33. 30198059 10.1007/s11154-018-9455-2 PMC6208575

[14] Annaiah TK , Kodakkattil S , Sriemevan A . Pregnancy with mitochondrial encephalopathy lactic acidosis and stroke‐like episodes (MELAS syndrome) leading to confusion in the diagnosis of pulmonary embolism. J Obstet Gynaecol 2007;27:618–619. 17896266 10.1080/01443610701546318

[15] Moriarty KT , McFarland R , Whittaker R , et al. Pre‐eclampsia and magnesium toxicity with therapeutic plasma level in a woman with m.3243A>G melas mutation. J Obstet Gynaecol 2008;28:349. 18569490 10.1080/01443610802048545

[16] de Laat P , Fleuren LH , Bekker MN , et al. Obstetric complications in carriers of the m.3243A>G mutation, a retrospective cohort study on maternal and fetal outcome. Mitochondrion 2015;25:98–103. 26455484 10.1016/j.mito.2015.10.005

[17] Say RE , Whittaker RG , Turnbull HE , et al. Mitochondrial disease in pregnancy: a systematic review. Obstetric Med 2011;4:90–94. 10.1258/om.2011.110008 PMC4989604 27579099

[18] Shi H , Waldman G , Tobochnik S , et al. Clinical reasoning: refractory status epilepticus in a primigravida. Neurology 2019;92:968–972. 31085725 10.1212/WNL.0000000000007507 PMC9245926

[19] Ojajarvi P . The adolescent Finnish child: a longitudinal study of the anthropometry, physical development, and physiological changes during puberty. Helsinki, Finland: University of Helsinki, 1982.

[20] Lindgren GW , Degerfors IL , Fredriksson A , et al. Menarche 1990 in stockholm schoolgirls. Acta Paediatr Scand 1991;80:953–955. 1755302 10.1111/j.1651-2227.1991.tb11758.x

[21] Juul A , Teilmann G , Scheike T , et al. Pubertal development in Danish children: comparison of recent European and US data. Int J Androl 2006;29:247–255. 16466546 10.1111/j.1365-2605.2005.00556.x

[22] Christensen KY , Maisonet M , Rubin C , et al. Progression through puberty in girls enrolled in a contemporary British cohort. J Adolesc Health 2010;47:282–289. 20708568 10.1016/j.jadohealth.2010.02.005 PMC5578456

[23] Bruserud IS , Roelants M , Oehme NHB , et al. References for ultrasound staging of breast maturation, tanner breast staging, pubic hair, and Menarche in Norwegian girls. J Clin Endocrinol Metab 2020;105:1599–1607. 10.1210/clinem/dgaa107 PMC7275631 32140730

[24] Martí‐Henneberg C , Vizmanos B . The duration of puberty in girls is related to the timing of its onset. J Pediatr 1997;131:618–621. 9386670 10.1016/s0022-3476(97)70073-8

[25] Mul D , Fredriks AM , van Buuren S , et al. Pubertal development in The Netherlands 1965–1997. Pediatr Res 2001;50:479–486. 11568291 10.1203/00006450-200110000-00010

[26] Aksglaede L , Sørensen K , Petersen JH , et al. Recent decline in age at breast development: the Copenhagen Puberty Study. Pediatrics 2009;123:e932–e939. 19403485 10.1542/peds.2008-2491

[27] Júlíusson PB , Roelants M , Eide GE , et al. Growth references for Norwegian children. Tidsskr Nor Laegefore 2009;129:281–286. 10.4045/tidsskr.09.32473 19219092

[28] Pagnamenta AT , Taanman JW , Wilson CJ , et al. Dominant inheritance of premature ovarian failure associated with mutant mitochondrial DNA polymerase gamma. Human Reprod 2006;21:2467–2473. 10.1093/humrep/del076 16595552

[29] Luoma P , Melberg A , Rinne JO , et al. Parkinsonism, premature menopause, and mitochondrial DNA polymerase gamma mutations: clinical and molecular genetic study. Lancet (London, England). 2004;364:875–882. 10.1016/S0140-6736(04)16983-3 15351195

[30] Majewska MD , Harrison NL , Schwartz RD , et al. Steroid hormone metabolites are barbiturate‐like modulators of the GABA receptor. Science 1986;232:1004–1007. 2422758 10.1126/science.2422758

[31] Wong M , Moss RL . Long‐term and short‐term electrophysiological effects of estrogen on the synaptic properties of hippocampal CA1 neurons. J Neurosci 1992;12:3217–3225. 1353794 10.1523/JNEUROSCI.12-08-03217.1992 PMC6575649

[32] Cramer JA , Gordon J , Schachter S , Devinsky O . Women with epilepsy: hormonal issues from menarche through menopause. Epilepsy Behav 2007;11:160–178. 17662661 10.1016/j.yebeh.2007.03.007

[33] Rupprecht R , Hauser CA , Trapp T , Holsboer F . Neurosteroids: molecular mechanisms of action and psychopharmacological significance. J Steroid Biochem Mol Biol 1996;56:163–168. 8603037 10.1016/0960-0760(95)00233-2

[34] Majewska MD . Neurosteroids: endogenous bimodal modulators of the GABAA receptor. Mechanism of action and physiological significance. Prog Neurobiol 1992;38:379–395. 1349441 10.1016/0301-0082(92)90025-a

[35] Herzog A.G . Hormonal changes in epilepsy. Epilepsia 1995;36:323–326. 7607109 10.1111/j.1528-1157.1995.tb01004.x

[36] Yanagawa T , Sakaguchi H , Nakao T , et al. Mitochondrial myopathy, encephalopathy, lactic acidosis, and stroke‐like episodes with deterioration during pregnancy. Intern Med 1998;37:780–783. 9804089 10.2169/internalmedicine.37.780

[37] Ormazabal A , Casado M , Molero‐Luis M , et al. Can folic acid have a role in mitochondrial disorders? Drug Discov Today 2015;20:1349–1354. 26183769 10.1016/j.drudis.2015.07.002

[38] Chou YF , Huang RF . Mitochondrial DNA deletions of blood lymphocytes as genetic markers of low folate‐related mitochondrial genotoxicity in peripheral tissues. Eur J Nutr 2009;48:429–436. 19437061 10.1007/s00394-009-0031-0

[39] Chou YF , Yu CC , Huang RF . Changes in mitochondrial DNA deletion, content, and biogenesis in folate‐deficient tissues of young rats depend on mitochondrial folate and oxidative DNA injuries. J Nutr 2007;137:2036–2042. 17709439 10.1093/jn/137.9.2036

[40] Fenech M . Folate (vitamin B9) and vitamin B12 and their function in the maintenance of nuclear and mitochondrial genome integrity. Mutat Res 2012;733:21–33. 22093367 10.1016/j.mrfmmm.2011.11.003

[41] Nikkanen J , Landoni JC , Balboa D , et al. A complex genomic locus drives mtDNA replicase POLG expression to its disease‐related nervous system regions. EMBO Mol Med 2018;10:13–21. 29109127 10.15252/emmm.201707993 PMC5760859

[42] Engelsen BA , Tzoulis C , Karlsen B , et al. POLG1 mutations cause a syndromic epilepsy with occipital lobe predilection. Brain 2008;131(Pt 3):818–828. 18238797 10.1093/brain/awn007

[43] Tzoulis C , Neckelmann G , Mork SJ , et al. Localized cerebral energy failure in DNA polymerase gamma‐associated encephalopathy syndromes. Brain 2010;133(Pt 5):1428–1437. 20400524 10.1093/brain/awq067

[44] Hikmat O , Eichele T , Tzoulis C , Bindoff LA . Understanding the epilepsy in POLG related disease. Int J Mol Sci 2017;18:1845. 10.3390/ijms18091845 PMC5618494 28837072

