# Supplementary material for: The impact of gender, puberty, and pregnancy in patients with POLG disease
Source: Ann Clin Transl Neurol. 2020 Sep 18;7(10):2019–25. doi: 10.1002/acn3.51199 (PMC7545595; doi:10.1002/acn3.51199)
Supplement: Supplementary file 1 — File S1. Timing of thelarche and menarche in the population of each participating country in the study. [file ACN3-7-2019-s001.pdf]

Supplementary file 1: Timing of thelarche and menarche in the population of each participating country in the study.

**Country[(ref)]**	**Study year**	**Mean Age of thelarche onset in years**	**Mean Age of menarche onset in years**
Norway [(1)]	2016	10.38	12.7
Norway[(2)]	2003-2006	-	13.3
Denmark[(3)]	2006-2008	9.86	13.13
Denmark[(4)]	1991-1993	10.88	13.42
Sweden[(5)]	1991	-	13.19
Finland[(6)]	1982	10.8	12.2
England, UK[(7)]	1999-2005	10.12	12.9
Nederland[(8)]	1996-1997	10.72	13.5
Spain[(9)]	1987	10.6	12.6

References:

1. Bruserud IS, Roelants M, Oehme NHB, Madsen A, Eide GE, Bjerknes R, et al. References for Ultrasound Staging of Breast Maturation, Tanner Breast Staging, Pubic Hair, and Menarche in Norwegian Girls. The Journal of clinical endocrinology and metabolism. 2020;105(5):1599-607.
2. Júlíusson PB, Roelants M, Eide GE, Moster D, Juul A, Hauspie R, et al. [Growth references for Norwegian children]. Tidsskrift for den Norske laegeforening : tidsskrift for praktisk medicin, ny raekke. 2009;129(4):281-6.
3. Aksglaede L, Sørensen K, Petersen JH, Skakkebaek NE, Juul A. Recent decline in age at breast development: the Copenhagen Puberty Study. Pediatrics. 2009;123(5):e932-9.
4. Juul A, Teilmann G, Scheike T, Hertel NT, Holm K, Laursen EM, et al. Pubertal development in Danish children: comparison of recent European and US data. International journal of andrology. 2006;29(1):247-55; discussion 86-90.
5. Lindgren GW, Degerfors IL, Fredriksson A, Loukili A, Mannerfeldt R, Nordin M, et al. Menarche 1990 in Stockholm schoolgirls. Acta paediatrica Scandinavica. 1991;80(10):953-5.
6. Ojajarvi P. The adolescent Finnish child: a longitudinal study of the anthropometry, phsical development, and physiological changes during puberty. Helsinki, Finland: University of Helsinki 1982.
7. Christensen KY, Maisonet M, Rubin C, Holmes A, Flanders WD, Heron J, et al. Progression through puberty in girls enrolled in a contemporary British cohort. The Journal of adolescent health : official publication of the Society for Adolescent Medicine. 2010;47(3):282-9.
8. Mul D, Fredriks AM, van Buuren S, Oostdijk W, Verloove-Vanhorick SP, Wit JM. Pubertal development in The Netherlands 1965-1997. Pediatric research. 2001;50(4):479-86.
9. Martí-Henneberg C, Vizmanos B. The duration of puberty in girls is related to the timing of its onset. The Journal of pediatrics. 1997;131(4):618-21.
