# Supplementary material for: The impact of gender, puberty, and pregnancy in patients with POLG disease
Source: Ann Clin Transl Neurol. 2020 Sep 18;7(10):2019–25. doi: 10.1002/acn3.51199 (PMC7545595; doi:10.1002/acn3.51199)
Supplement: Supplementary file 2 — File S2. Major clinical features stratified according to the gender and puberty onset. [file ACN3-7-2019-s002.pdf]

Supplementary file 2. Major clinical features stratified according to the gender and puberty-onset.

	All	All Males	All females	Onset prior to 12 years males	Onset prior 12 years females	Onset after 12 years males	Onset after 12 years females
**Seizures**	69%(n=107/155)	70%(n=53/76)	68%(n=54/79)	57%(n=35/62)	43%(n=27/62)	41%(n=18/44)	59%(n=26/44)
**SLE**	37%(n=52/139)	34%(n=23/67)	40%(n=29/72)	73%(n=16/22)	27%(n=6/22)	27%(n=7/26)	73%(n=19/26)
**Ataxia**	63%(n=87/138)	62%(n=41/66)	64%(n=46/72)	59%(n=13/22)	41%(n=9/22)	44%(n=25/57)	56%(n=32/57)
**PNP**	53%((n=65/123)	52%(n=31/60)	54%(n=34/63)	50%(n=4/8)	50%(n=4/8)	50%(n=26/52)	50%(n=26/52)
**Hepatopathy**	64%(n=96/151)	63%(n=46/73)	64%(n=50/78)	54%(n=32/59)	46%(n=27/59)	36%(n=10/28)	64%(n=18/28)
**Ptosis**	34%(n=51/148)	38%(n=28/73)	31%(n=23/75)	50%(n=4/8)	50%(n=4/8)	55%(n=22/40)	45%(n=18/40)
**PEO**	39%(n=56/144)	41%(n=29/71)	37%(27/73)	25%(n=1/4)	75%(n=3/4)	54%(n=26/48)	46%(n=22/48)

SLE: stroke like episodes, PNP: peripheral Neuropathy, PEO: progressive external ophthalmoplegia.
